# Supplementary material for: In Vitro and In Silico Analysis of Epithelial-Mesenchymal Transition and Cancer Stemness as Prognostic Markers of Clear Cell Renal Cell Carcinoma
Source: Cancers (Basel). 2023 May 1;15(9):2586. doi: 10.3390/cancers15092586 (PMC10177434; doi:10.3390/cancers15092586)
Supplement: Supplementary file 1 [file cancers-15-02586-s001.zip › cancers-2330309-supplementary.pdf]

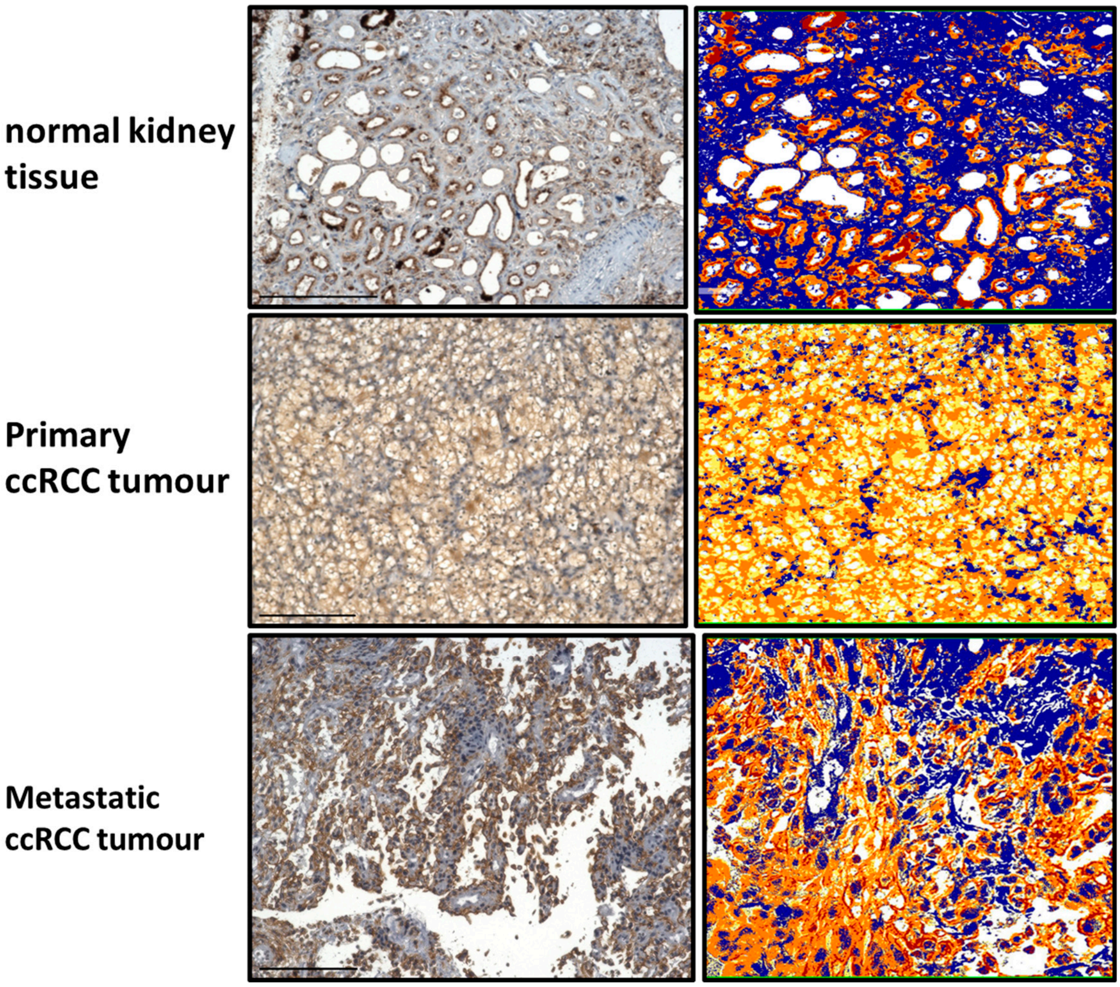


**Figure S1.** Visualization of positive pixel count of CD44 staining for the scanned brown color image analysis by Aperio ImageScope in normal kidney tissue, primary ccRCC tumour and metastatic ccRCC tumour.
